# The gender gap in commenting: Women are less likely than men to comment on (men’s) published research

**DOI:** 10.1371/journal.pone.0230043

**Published:** 2020-04-01

**Authors:** Cary Wu, Sylvia Fuller, Zhilei Shi, Rima Wilkes

**Affiliations:** 1 Department of Sociology, York University, Toronto, Ontario, Canada; 2 Department of Sociology, University of British Columbia, Vancouver, British Columbia, Canada; 3 School of Public Administration, Zhongnan University of Economics and Law, Wuhan, China; Universidad de las Palmas de Gran Canaria, SPAIN

## Abstract

Subtle gender dynamics in the publishing process involving collaboration, peer-review, readership, citation, and media coverage disadvantage women in academia. In this study we consider whether commenting on published work is also gendered. Using all the comments published over a 16-year period in *PNAS* (N = 869) and *Science* (N = 481), we find that there is a gender gap in the authorship of comment letters: women are less likely than men to comment on published academic research. This disparity is greater than gender differences in the publication of research articles. There is also a gendered pattern in commenting: women comment writers are relatively less likely to engage with men’s research. If left unaddressed, these patterns in academic commenting could impede scholarly exchange between men and women and further marginalize women within the scientific community.

## Introduction

A growing body of research shows that women are disadvantaged across the stages of academic publishing, including collaboration, peer-reviewing, readership, citation, and media coverage. Men are more likely to collaborate with men than with women and women are given less credit when collaborating with men [1–3]. Women are less likely to be the first or last author on articles published in prestigious journals [4,5], women’s research is less likely to be read, shared, and cited [4,6,7] for alternative perspectives, please see [8,9], women are held to higher peer review standards and hence female-authored papers take half a year longer to publish [10], women are less likely to be invited to submit papers for journals and to act as reviewers [11–13], men are less likely to respond to requests by women editors to review papers [14, 15], and women’s research is less likely to receive media coverage [16,17]. These differences matter—their combined effect make it harder for women scholars to get jobs, advance in their careers, and ultimately, to attain scientific eminence [18–23] for alternative perspectives, see [24–26].

In this study, we explore whether there is a gender gap in commenting on published work. Many leading general scientific research journals including *Science*, *Nature*, and *PLOS One* publish comments as do leading disciplinary journals across a variety of fields including *Physical Review Letters*, *American Sociological Review*, and *American Economic Review*. The comment letters that appear in these journals allow readers to constructively point out flaws, discrepancies and differences of opinion vis-a-vis previously published research. While the practice of commenting on published work plays an essential role in promoting scholarly discussion, knowledge exchange, and scientific advancement [27,28], this career-building activity has received little scholarly attention. If women comment less often than men, this could contribute to lower levels of overall academic visibility (e.g., see [4,21,28,29]).

To study commenting we consider comments published in two leading journals—*PNAS* and *Science*. Both are among the world’s most comprehensive, high impact, and widely read scientific journals. *Science* has published comments since 2003, while *PNAS*’s weekly comment letters date back to 2007. After showing that women do, indeed, comment less often than men, we test a number of explanations about the meaning of such a gap and whether, and in what way, it is “gendered”. Is the heart of the problem that women comment less because there are fewer women available to comment? Is the issue that women are in positions that depress willingness to take a risk? Perhaps women are generally more risk averse, or are differentially punished for taking a risk? Or are women more concerned about the consequences of commenting for those they target?

## Explaining gender gaps in commenting

Why might women comment less often than men? One possibility is that commenting is about *Research Practice*. While quantity and quality of publication remains the “gold standard” by which academic careers are judged [30], there is heterogeneity in how these are assessed within specific fields and disciplines. In economics, for example, it is common to publish working papers, and these can be cited more often than the corresponding publication [31]. Some fields are more willing to consider open access publication than others [32] and some disciplines value books [33] more than journal articles. Perhaps commenting is simply more likely in some fields than others (see also [8] on field differences in publication). Accordingly, women’s lower rates of publishing comments could represent their relative under-representation in the fields that tend to comment.

Another possibility is that commenting is about challenge, risk, and reward. To understand how and why risk, challenge and reward could be reflective of gender, and in what ways, requires considering not only the characteristics of the comment author but also the characteristics of the author of the article they are commenting on (the target).

### General risk aversion

Public commentary explicitly challenges research recognized as authoritative by virtue of its publication in a high-prestige journal. As such, it requires a willingness to assert the superiority of one’s own scholarly insights above those of someone else. While engaging in high profile scholarly debate can build one’s reputation, publicly criticizing someone’s work is also a potentially high-risk form of scholarly contribution and engagement. The general risks include damage to one’s own reputation (if one’s critique is perceived as invalid, unfair, or inappropriately motivated) and, potentially, retaliation in either the short or long term. For these reasons, scholars might delay commenting until they are more established in their career. In this case a gender difference in commenting may be attributed to the fact that women are concentrated in more junior positions (see e.g., [18, 23] on gender distribution of academic positions). In this case, a difference in commenting represents a pipeline effect. At similar career levels, we should not see gender differences in commenting.

### Gendered risk aversion

It could be that gender shapes scholars’ weighing of the relative risks and rewards associated with commenting. Decades of research have shown that men and women often behave differently in situations involving risk taking [34–37]. Men tend to be more willing to engage in high risk behaviours [38]. If women are more risk averse, then they should be more concerned about the negative consequences of publicly challenging someone who can retaliate. If this is true, in addition to depressing women’s commenting rates overall, gendered disparities in commenting should be stronger where risk to career is more salient, such as when authors do not have a permanent position. We would also expect weaker gender differences in commenting where the target article is authored by a more junior person, and hence where the risks of opposing them are lower.

### Gendered risk

Another possibility is that the consequences of commenting are gendered. Women and men are differently penalized and rewarded for the same behavior. Gendered cultural expectations that women are communal and not assertive [39,40] clash with the agentic, critical stance of commenting. Such gender stereotypes are not simply descriptive (indexing beliefs about men and women’s characteristics), but also reflect cultural beliefs about how men and women *should* (or should not) behave. Violating gender stereotypes tends to elicit disapproval [36,41–43]. Thus while men may be positively valued for the assertive and competitive act of commenting insofar as it is congruent with masculine stereotypes, women potentially pay a priceIndeed, when women are perceived as behaving assertively, and demonstrate success in male-dominated arenas, their “likability” typically diminishes with negative social and economic consequences [36,41–43]. To the extent that scholarly opportunities (e.g. for collaboration) are impacted by perceptions of likability, this could prove particularly harmful for women. Violations of gender stereotypes may elicit sanctions not simply because they challenge norms, but also the status hierarchies that undergird them [41, 44]. While commenting in general could be disproportionately risky for women, this should be more pronounced when the target is a male scholar insofar as this challenges the presumptive superiority of men. This implies that, relative to male commentators, women will be less likely to target articles authored by men.

### Gendered caring depresses challenge

Since commenting entails publicly challenging (and to a certain extent taking down) others, gendered norm of caring may also come into play. Commenting is not necessarily a nice practice, as it calls into question someone else’s work. Even though commented-upon papers tend to end up as journals’ most cited papers [45], and hence being targeted may ultimately be beneficial, this may not be common knowledge. It is reasonable to think that potential comment writers might worry about their comment leading to negative consequences for the person(s) targeted. Apart from high-risk altruism, women tend to report being more caring [46]. While some argue this is an innate difference, others suggest it results from social processes enforcing gendered expectations [47, 48]. Irrespective of the cause, this could depress women’s rates of commenting as well as their likelihood of targeting those deemed vulnerable—in the academic world this is junior scholars.

## Data and coding strategy

To study patterns of commenting we use author information from all comment letters and corresponding research articles published in *Proceeding of the National Academy of Sciences of the United States of America (PNAS)* and *Science*. Both journals are multi-disciplinary, which makes it possible to consider field-specific differences in commenting patterns. Specifically, we created a dataset pairing research papers and their comment letters from both *PNAS* and *Science*. The raw data is available from (weblink to dataset here).

PNAS: The data come from *PNAS*’ journal archive *Letters and Replies* (see http://www.pnas.org/). We obtain information from the journal archives, and by searching for online author profiles. To code, for example, the author information of the letter “Age-aggregation bias in mortality trends” (Andrew Gelman and Jonathan Auerbach; February 16, 2016 vol. 113 no. 7), we obtain the name of the first author (Andrew Gelman) and the author affiliation (Department of Statistics, Columbia University, New York, NY 10027, as well as the number of authors and corresponding author status from the journal archive. Combining the name and the affiliation, we searched online for the author’s profile, curriculum vitae and pictures to assess the gender (male), and current academic rank (full professor) as well as the research field (e.g. social sciences). We collect the same information for the last author. We collected the same information for the author of the corresponding research paper “Increased mortality for white middle-aged Americans not fully explained by causes suggested” (Anne Case and Angus Deaton; February 16, 2016 vol. 113 no. 7). All the author information was identified manually and cross-validated by at least two authors and/or research assistants.

Science: We collect data from *Science Magazin*e’s online article archive using the search function (http://www.sciencemag.org/). Because all comments published in *Science* start with “comment on” in the title, we use key words “comment on” with additional criteria 1) limit search to “Title”, 2) source “Science”, 3) article type “Perspectives and commentary” (see Figure Science) to collect all comments published in *Science*. To code the information for each specific comment, we follow the same approach as described in coding the PNAS comments and articles.

We searched all comments published in both *PNAS* and *Science* from the beginning of their publication of comments to the most current issue at the time of data collection. There were 869 comment letters referring to 762 journal articles between December 18, 2007 and September 25, 2018 from *PNAS* and 481 comment letters referring to 474 journal articles from *Science* between January 1, 2003 and December 31, 2017. In total, we obtained 1,350 comment letters referring to 1,236 research articles. In some cases, multiple comment letters targeted the same research paper. We must acknowledge that, both *PNAS* and *Science* are top ranked high prestige high stakes journals that confer heightened visibility and can make or break a career. Their published articles and comments might not be representative of patterns of commenting in all journals, where the stakes are lower.

## Results

**Fig 1:**
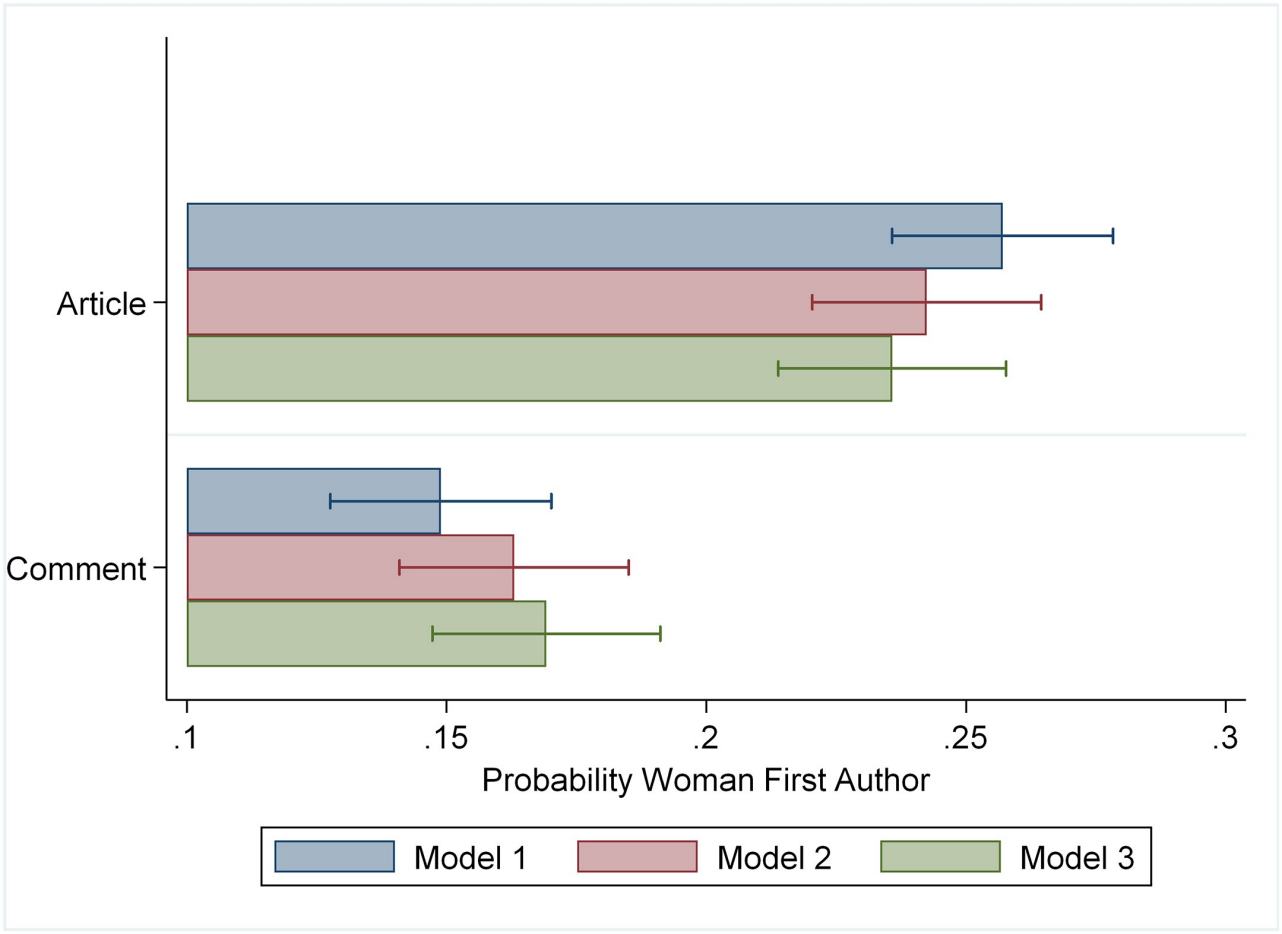
Estimated probability first author is a woman by publication type.

We begin by assessing whether there is an overall gender difference in academic commenting. We focus on the gender of the person taking primary responsibility for the comment, the first author. To see whether this pattern simply reflects women’s lower overall rates of publication in *PNAS* and *Science*, we estimate a linear probability model predicting women’s authorship by type of publication. Our baseline model (M1) without controls reveals that overall only 15% of the comments have a female first author versus 26% of articles, a significant difference (p < .001). Fig 1 graphs the estimated proportion of articles and comments with female first authors for this and subsequent models with various controls—full regression results are in Table 1.

**Table 1:** Linear probability estimates that first author is a woman.

	Model 1	Model 2	Model 3	Model 4
Comment (Article)	-0.108***	-0.079***	-0.067***	-0.108***
	(.0150)	(.0170)	(.0170)	(.0290)
Year		0.004	0.004	0.004
		(.0020)	(.0020)	(.0020)
Journal		-0.065***	-0.054**	-0.051**
		(.0180)	(.0180)	(.0180)
*Number of authors (1)*				
2		0.055*	0.048	0.051
		(.0260)	(.0260)	(.0260)
3		0.073**	0.065*	0.067*
		(.0270)	(.0270)	(.0270)
4		0.113***	0.097**	0.101***
		(.030)	(.030)	(.030)
5		0.039	0.026	0.029
		(.0340)	(.0340)	(.0340)
6+		0.126***	0.107***	0.109***
		(.0270)	(.0270)	(.0270)
*Field (Biological Sciences)*				
Physical Sciences			-0.065***	-0.064***
			(.0190)	(.0190)
Social Sciences			0.032	0.034
			(.0230)	(.0230)
First Author is Corresponding			-0.109***	-0.133***
			(.0170)	(.0220)
Comment # First Author is Corresponding				0.058
				(.0340)
Constant	0.257***	-6.913	-7.197	-7.471
	(.0110)	(4.4420)	(4.4080)	(4.410)
R-squared	0.018	0.037	0.056	0.057
Standard errors in parentheses				

* p<0.05,

** p<0.01,

*** p<0.001

If women’s authorship differs in comments versus articles, this suggests that gender differences in commenting cannot simply be attributed to factors that depress women’s publication in *PNAS* and *Science* generally. In what follows, we focus on this difference in women’s probability of being the first author of comments versus articles as the most relevant indicator of a gender gap in commenting. Model 2 adds a suite of basic controls to account for potential differences—year, journal, and number of authors. The difference in female authorship between comments and articles shrinks slightly, but remains substantial and significant (p < .001).

We next add field (biological, physical, or social science), and corresponding author status (Model 3). The field covariates account for differences tied to women’s underrepresentation in fields with higher rates of commenting, testing our argument about differences in *research practice*. Corresponding author status is included to proxy differences attributable to aversion to the risks of commenting among those in the most vulnerable positions (Model 4)–our argument about *general risk aversion*. Ideally, we would have detailed data on position at the time of publication to account for gradations of risk across academic hierarchies. However, this is difficult to ascertain from publically available data for the authors in our study. First authors who are not also the corresponding author in multi-author publications provide a reasonable approximation of those who do not have permanent positions, and who are therefore the most vulnerable. We surmise that a first author who does not place her/himself as the corresponding author likely does not have a permanent job (i.e. a graduate student or postdoctoral fellow). Such authors may anticipate a change in their institutional affiliation that would make corresponding author information outdated, and/or because academic norms tend to encourage the person ultimately most responsible for the research be designated as corresponding author. To our knowledge there is no direct evidence in the research literature on how authors make decisions about corresponding author status. The corresponding author tends to be viewed as the person taking full responsibility for a paper, at least in some fields, but the limited research available also reveals variability in understandings about its meaning [49]. There is evidence that when first authors are not the corresponding author, raters perceive them as playing less of a leadership role in the research [50,51,52]. We therefore expect that first authors will prefer to retain the role of corresponding author unless there is a compelling reason to act otherwise [53]. For students and postdocs, this may be the research supervisor.

Commenting continues to be associated with significantly lower female authorship than article publication. Field effects, moreover, do not contribute to this gap. Assessing this entails testing differences in coefficients between nested models with the other controls that do and do not include the covariates of interest. For convenience, we implement this test with the khb module in stata. While corresponding author status does partially explain the difference in female authorship in comments versus articles, its role is small, decreasing the gap in proportion female by only .012.

**Fig 2:**
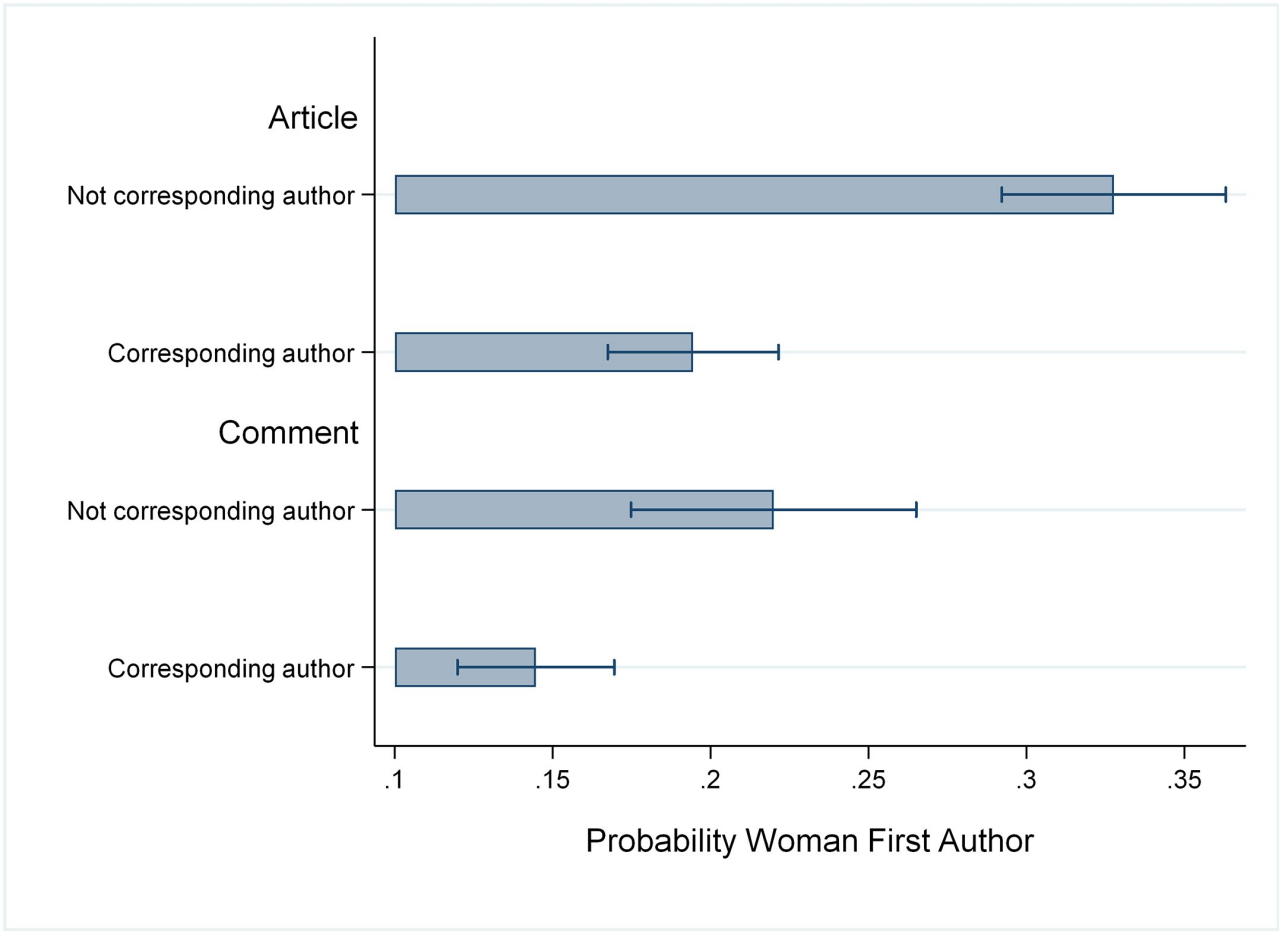
Estimated probability first author is a woman by publication-type and corresponding-author status.

We can shed some light on the persuasiveness of arguments about *gendered risk aversion* by first seeing if being in a more vulnerable academic position increases the gender gap in commenting vs article authorship. Fig 2 depicts the proportion of women authors in comments and articles by this status, with full model results presented in Model 4.

There is a higher rate of female authorship of both comments and articles among the vulnerable/junior scholars, likely because women have higher representation in lower academic ranks. While the gender gap in commenting versus article authorship is indeed wider among those who are not corresponding authors as the gendered risk aversion argument predicts, this difference is not significant at conventional levels (p = .09).

If greater risk aversion drives women’s lower rates of commenting, we would also expect a smaller gender gap in comment authorship where the targeted first author is relatively powerless as this should make commenting risks less salient generally. Conversely, if women are commenting less overall because they are more concerned about harming the careers of others (*gendered caring depresses challenge*), the gender gap in commenting in favour of men will be greater when the target is more vulnerable. To assess this, we focus on comment authorship only, estimating a linear probability models testing whether gender of comment author impacts the likelihood that the first author of the target article is not also a corresponding author. Model 5 in Table 2 presents these results. In fact, gender differences in the probability of targeting such articles are small and not statistically significant. Results thus suggest that women’s differing orientation to risk per se (whether to themselves or targets) is unlikely to drive their lower rates of commenting.

**Table 2:** Linear probability estimates that first author of target article is corresponding author.

	Model 5
Woman comment author	-0.011
	(.0360)
Year	0.009*
	(.0040)
Journal	0.151***
	(.0320)
*Field (Biological Sciences)*	
Physical Sciences	0.063
	(.0320)
Social Sciences	0.254***
	(.0390)
*Number of comment authors (1)*	
2	-0.003
	(.0360)
3	-0.008
	(.0390)
4	-0.071
	(.0450)
5	-0.172**
	(.0580)
5+	-0.106*
	(.0440)
*Number of article authors (1)*	
2	-0.039
	(.0730)
3	-0.09
	(.0730)
4	-0.147*
	(.0740)
5	-0.113
	(.0770)
5+	-0.162*
	(.070)
Constant	-16.620*
	(7.5570)
R square	0.073
Standard errors in parentheses	

* p<0.05,

** p<0.01,

*** p<0.001

Findings thus far suggest that neither differences in men and women’s general orientation to risk or women’s disproportionately vulnerable academic status (alone or in tandem) explain women’s lower rates of commenting. Perhaps the risks faced by women differ in gendered ways, such that the consequences of commenting are potentially more negative for women. Recall that arguments about gendered risks imply that not only should commenting in general be disproportionately risky for women in light of prescriptive gender stereotypes, but that this should be more pronounced when the target is a male scholar (insofar as this challenges the presumptive superiority of men). A linear probability model predicting the gender of the first author of the targeted article by the gender of the first author of the comment assesses this argument (Model 6, Table 3).

**Table 3:** Linear probability estimates that first author of targeted article is a man.

	Model 6	Model 7
Woman comment author	-0.099**	-0.081*
	(.0330)	(.040)
Year	-0.002	-0.002
	(.0030)	(.0030)
Journal	0.079**	0.079**
	(.0290)	(.0290)
*Field (Biological Sciences)*		
Physical Sciences	0.097**	0.095**
	(.0290)	(.0310)
Social Sciences	-0.011	0.012
	(.0350)	(.0390)
Woman # Physical Sciences		0.019
		(.0910)
Woman # Social Sciences		-0.14
		(.0930)
*Number of Comment Authors (1)*		
2	-0.006	-0.004
	(.0330)	(.0330)
3	-0.009	-0.005
	(.0360)	(.0360)
4	-0.025	-0.024
	(.0410)	(.0410)
5	-0.052	-0.054
	(.0530)	(.0540)
5+	0.023	0.024
	(.0410)	(.0410)
*Number of Article Authors (1)*		
2	-0.071	-0.076
	(.0670)	(.0670)
3	-0.103	-0.105
	(.0670)	(.0670)
4	-0.119	-0.122
	(.0680)	(.0680)
5	-0.026	-0.028
	(.0710)	(.0710)
5+	-0.176**	-0.180**
	(.0640)	(.0640)
Constant	5.053	4.734
	(6.9320)	(6.9350)
R squared	0.043	0.044
Standard errors in parentheses		

* p<0.05,

** p<0.01,

*** p<0.001

While both men and women are more likely to target articles first authored by men, this is significantly more pronounced among men. Since women are under-represented among *PNAS* and *Science* article authors, this would, in itself, depress women’s overall rates of commenting relative to men.

Note that it is not possible to ascertain whether this dynamic is driven by women privileging other women as targets (as the gendered risk argument asserts), or men disproportionately privileging other men (or some combination thereof). Cut-throat competition among men in masculinity contests at work can effectively sideline and marginalize women [54]. On the other hand, men might enjoy masculinity contests. This is because their pursuit of ‘success and winning’ could boost up their sense of mastery and achievement which in turn yields higher psychological wellbeing [55]. It may be that commenting reflects a genteel variation of this dynamic, with men focusing on other men as the targets most worthy of engagement.

It is also possible that women are less likely than men to target articles written by men not because of differential consequences, but because their expertise is more relevant with respect to work undertaken by women. We have classified articles and comments by broad fields in this study, but there can be pronounced differences in men and women’s representation across different disciplines within these. In the social sciences, for example, women with doctorates are overrepresented in Psychology but underrepresented in Economics [56]. Overall, women’s representation varies less across the fields within the biological sciences than it does across social or physical science disciplines (*ibid)*. If such differences drive gender matching in comments/articles, matching should be less pronounced in the biological sciences. To test this, we add an interaction between field and author gender to our model predicting gender of target article author (Model 7 Table 3). The interaction is not significant (and is positive for the physical sciences). While this does not support the presumption that gender matching is driven by sub-field gender segregation, we should nonetheless be cautious in our interpretation given the possibility of substantial gender segregation in specialization areas even within disciplines [5, 3].

Ultimately, we cannot directly test whether women suffer more negative consequences for challenging the status quo by commenting with our data. However, this remains a plausible explanation for their lower rates of commenting in light of the patterns we have uncovered and lack of support we find for the other arguments.

### Sensitivity tests

In all of our analyses, we focus on the first author of a comment or article as the most relevant indicator of authorship gender. However, in multi-authored publications a case could be made that other authorship positions matter. For example, insofar as status hierarchies make it riskier for women to comment on men’s research, the gender of the highest status author of the targeted article may also be relevant. Ascertaining the highest status author is challenging insofar as it would involve subjective and retrospective assessments of career stature at the time the article was published. The most senior author is easier to ascertain, although even here ambiguity remains given field differences in authorship conventions. In some fields, the last author is most senior, but this is not universal (for example, in Sociology, and hence in this article, authorship follows a pattern of descending order of contribution, as it does in Sociology generally), making it a problematic measure for interdisciplinary journals like *Science* and *PNAS*. A corresponding author who is not also first author is likely the most senior author, but corresponding authors who are also first authors are not necessarily the most senior. Recognizing that neither measure is perfect, we can at least ascertain the robustness of the general pattern with additional analyses that code gender in relation to the last author and the corresponding author. The same general pattern prevails (see S1 Fig). Women are less likely to be the last author and the corresponding author than are men, a pattern that is significantly more pronounced for comments than articles. The gender gap in commenting is, however, significantly smaller than when we focus on first authorship. Gender of neither the last nor corresponding author predicts the likelihood of targeting an article with a more vulnerable first author. The only case where we see a discrepancy is when predicting the gender of the author of the targeted article. There is no significant difference in men and women’s probability of targeting a male last-authored article when we measure gender of comment authorship by the last or corresponding author.

## Conclusion

Ultimately, this study finds that women are less likely to engage in academic commenting, a disparity that exceeds gendered differentials in article publication. The gap cannot be explained by variability in the field-specific gender-ratio. Nor can women’s over-representation in the positions most sensitive to career risks, or greater general sensitivity to risks to others explain the disparity. Women also direct a lower share of their comments towards men’s research than do men. Taken together, the two sets of findings are most consistent with the argument that women’s lower rates of commenting stem from specifically gendered costs to challenging authoritative research, especially when the challenge is to traditional status hierarchies. We caution, however, that we can only indirectly test this argument with our data.

Indeed, a limitation of our research is that we cannot differentiate whether our findings result from gender differences in the submission of comments or from editorial decisions about which comments to publish. In the latter case it is possible that women actually submit an equal proportion of comments but that those comments are less likely to be accepted for publication. We don’t see a strong prima-facie reason why editorial decisions would be more weighted against women authors of comments versus articles, but this is is an avenue worthy of future investigation. If women’s scholarship is viewed as less authoritative in light of gendered status differentials, their commentary may be seen as less credible, and thus less likely to be accepted for publication, particularly when they critique men’s work. Our findings with respect to the mechanisms underlying the gender differences we uncover should therefore best be understood as provisional, and as pointing to useful directions for future research.

Regardless of the mechanisms driving our findings, the basic empirical patterns are clear, and their impact is not trivial. Women’s lower rates of commenting mean they benefit less from participation in high-impact scholarly debate. The combination of gender matching and higher rates of male commenting further marginalizes women. Fewer articles written by women benefit from the enhanced visibility that comes with this type of scholarly engagement. These gender patterns in academic commenting could impede scholarly exchange between men and women and further marginalize women within the scientific community. Furthermore, when women experts are excluded, the academic community as a whole is deprived of fresh ideas and diverse opinions [29].

Problems of submission and/or acceptance rate differentials could be solved were editors to specifically promote more scholarly exchange between men and women. However, in the case of invited commentary, recent research suggests that women have 21% lower odds of authoring an invited commentary in medical journals compared with men with similar scientific expertise, seniority, and publication metrics [28]. Hence, editorial attention to the issue of gendered patterns is essential to address the gender bias in commenting more broadly. Editors are encouraged to specifically invite women to write comments [11], and especially comments on men’s published work. Insofar as commenting also increases the visibility of commented-upon research, this would also help counter barriers to women’s representation in high-impact scholarly debate.

## Supporting information

S1 Fig Difference in proportion of articles vs comments authored by women by comment first author, last author, and corresponding author status. * estimates from models controlling for field, publication year, journal, and number of authors.
(DOCX)

S1 Table Summary statistics of key variables in analysis. (DOCX)

S1 Data (DTA)
