# Correction: The gender gap in commenting: Women are less likely than men to comment on (men’s) published research

**DOI:** 10.1371/journal.pone.0232229

**Published:** 2020-04-17

**Authors:** The PLOS ONE Staff

There are errors in the Author Contributions. The publisher apologizes for the errors. The correct contributions are:

**Conceptualization:** Cary Wu, Sylvia Fuller, Rima Wilkes.

**Data curation:** Cary Wu, Zhilei Shi, Rima Wilkes.

**Formal analysis:** Cary Wu, Sylvia Fuller, Rima Wilkes.

**Writing–original draft:** Cary Wu, Sylvia Fuller.

**Writing–review & editing:** Cary Wu, Sylvia Fuller, Rima Wilkes.

There is an error in the Acknowledgments section. The correct statement is as follows: We gratefully acknowledge financial support from York University Libraries’ Open Access Author Fund. We also thank Nicole Malette, Narada Lobo, Emily Truong Cheung, Nick Chretien, May Chan, Yue Qian, Howard Ramos, and Malcolm Fairbrother for their assistance and helpful comments and advice.
