# Supplementary material for: The gender gap in commenting: Women are less likely than men to comment on (men’s) published research
Source: PLoS One. 2020 Apr 1;15(4):e0230043. doi: 10.1371/journal.pone.0230043 (PMC7112170; doi:10.1371/journal.pone.0230043)
Supplement: S1 Fig — * estimates from models controlling for field, publication year, journal, and number of authors. (DOCX) [file pone.0230043.s001.docx]

SI Figure A: Difference in proportion of articles vs comments authored by women by comment first author, last author, and corresponding author status

*Note: estimates from models controlling for field, publication year, journal, and number of authors
