# Supplementary material for: The gender gap in commenting: Women are less likely than men to comment on (men’s) published research
Source: PLoS One. 2020 Apr 1;15(4):e0230043. doi: 10.1371/journal.pone.0230043 (PMC7112170; doi:10.1371/journal.pone.0230043)
Supplement: S1 Table — (DOCX) [file pone.0230043.s002.docx]

S1 Table. Summary statistics of key variables in analysis

Note: There is some evidence that the size of the gender gap is a function of how it is measured, and depends on factors such as length of career, and cohort ment. For example, women have shorter careers so their annual productivity is the same; “Others find that this gender gap shrinks over the career trajectory and that it has largely disappeared or reversed in more recent cohorts, with women publishing more than men (van Arensbergenet al. 2012; Xie and Shauman 1998).” (cited in King et al 2017 p16) The gap is, of course larger in some places than others.
